# Effect of predictive value of progesterone level on the day of HCG injection for IVF success in women with infertility due to tubal factor or polycystic ovarian syndrome referred to the women hospital, Tehran, 2009

**Published:** 2012-07

**Authors:** Azizeh Ghaseminejad, Zahra Rezaee, Mitra Forootan, Taraneh Hosseinipoor, Forough Forghani, Pooneh Nikuei

**Affiliations:** 1 *Department of Obstetrics and Gynecology, Mirza Kouchakkhan Hospital, Tehran University of Medical Sciences, Tehran, Iran.*; 2 *Department of Obstetrics and Gynecology, Zabol University of Medical Sciences, Zabol, Iran.*; 3 *Hormozgan Fertility and Infertility Research Center, Hormozgan University of Medical Sciences, Bandarabbas, Iran.*

**Keywords:** *Serum progesterone Level*, *Infertility*, *PCOS*, *Tubal factor*, *IVF*

## Abstract

**Background:** Polycystic ovarian syndrome is one of the most common causes of endocrine disorders and main reason of infertility due to anovulation and recurrent abortions. Progesterone has been shown to have an important role in fertilization of oocyte and fetal implantation.

**Objective: **The purpose of this study was to compare the predictive value of progesterone level on IVF success in women with infertility due to tubal factor or PCOS.

**Materials and Methods:** In a stratified cohort study, we assigned 76 infertile women of 20-38 years old who referred to women hospital into two equal groups with fallopian tube factor infertility and PCOS. We measured the plasma levels of progesterone and estradiol on the day of HCG administration. The patients were divided into two groups based on progesterone level cut off point of 1.2ng/ml. Thereafter the incidence of pregnancy (chemical by β-HCG measurement and clinical by ultrasonography up to the 6 weeks after fetal transfer) was compared in these groups.

**Results:** Total pregnancy rates were 15.8% in patients with tubal factor infertility and 26.3% in women with PCOS. In women with PCOS, the pregnancy rate was less in patients with progesterone level <1.2 ng/ml. However this difference was not statistically significant. Likewise, we did not observe any significant differences in pregnancy rate in patients with fallopian tube factor infertility.

**Conclusion:** Serum progesterone level on the day of HCG administration is not well predictive of the IVF success in infertile women due to fallopian tube factor or PCOS. To obtain more uniform results, we recommend use of larger samples while the bias variable is taken into account and the ROC curve is used for determination of the unique serum progesterone level.

## Introduction

An ovulatory dysfunction is a common problem and is responsible for about 40% of female infertility. Polycystic ovarian syndrome (PCOS) remains one of its leading causes (1). It is a main reason of infertility due to anovulation and recurrent abortions, which could be considered as a risk factor for breast cancer (1-2). 

Clomiphene citrate is considered as the drug of choice for first line treatment of an ovulatory dysfunction (3), however, pregnancy rate is disappointingly lower than expected (50% or less). Individuals not responding to this treatment are often called Clomiphene Citrate Resistant (CCR). An alternative medication for CCR’s is traditionally administration of exogenous gonadothropine by injection. If this also fails the treatment for these cases is IVF (4). 

Most often GnRH analogs are used in conjunction with gonadothropine to prevent the possibility of spontaneous LH surge and ovulation prior to the egg retrieval. It is reported that, despite an effective suppression of endogenous gonadothropine by GnRH analog, there is a small increment in plasma progesterone up to 20% of stimulated cycles (5). Thus effect of progesterone increase on the cycle outcome is still controversial and not clear. 

While some authors reported negative effect on the pregnancy rate when the plasma level of progesterone is more than 0.9 ng/ml on the day of HCG injection in IVF cycles (6), others such as Hoffman and coworkers (7, 8) did not find any significant relationship between these two items. They also stated that the high level of plasma progesterone on the day of HCG injection on ovum donors has no adverse effect on pregnancy rate. In 1993, Lergo *et al* (9) showed that premature LH surge with elevated progesterone level increased pregnancy rate of ovum donors during IVF cycles. 

The aim of this study is evaluate the effect of plasma level of progesterone on the day of HCG injection on rate of pregnancy in IVF cycles of PCOS cases.

## Materials and methods

This stratified cohort study was conducted in a period of one year (2009) on 76 infertile women with PCOS and tubal factor who were suitable candidates for the IVF program. The certification of medical ethic committee was approved by tehran university of medical sciences. All of these PCOS cases were between 20-38 years. They were evaluated for other causes of infertility with HSG, laparoscopy. Their partner’s sperm analyses according to the guideline of WHO were all normal. 

The criteria for selection of PCOS patients were based on: 1) A history of amenorreah or oligomenorreah, 2) Hyperandrogenism symptoms, eg. Hirsutism according to Ferriman Gallweg score that was greater than seven, 3) Elevated LH or LH/FSH >2, and 4) Increased ovarian volume more than 9 ml or antral follicle count of 10 or more in ultrasound imaging. Infertile women with tubal factor defined as obstruction of both tubes which documented by laparascopy and have been candidate for IVF and aged between 20-38 years. 

Excluding criteria were under 20 and over 38 years old, not candidates for IVF, other causes of infertility than tubal factor and PCOS, Male factor presence that needs TESE. All patients underwent long pituitary down regulation protocol. They were given low dose of OCP pill on the third day of menstruation then from 20-21 day of menstrual cycle (late luteal phase) GnRH agonist (Superfact Hocchest Aquila, Italy) 500 microgram per day sub cutaneous started.

After two weeks of administering superfact dose or the third day of next menstruation cycle, serum estradiol and vaginal sonographic scan were performed to confirm pituitary suppression. If serum estradiol level was below 50 Picogram/ml and there was no follicle or cyst greater than 10 mm and endometrium thickness was less than 4 mm induction ovulation would begin. Otherwise, GnRH agonist would be given for an extra week up to a maximum of 21 days. 

150-225 unit gonadothropin [rFSH (gonal-f; Serono, Aubonne, Switzerland)] according to age, weight and FSH level on the third day of menstruation started. The superfact decreased to half of its initial dose on that day. After a period of 5 days of gonadothropin treatment, the dose of gonal-f was regulated depending on ovarian response that was monitored by vaginal ultrasound. When there were at least two 18-mm follicles, HCG (Profasi, serono, Rome, Italy) 10000 IU was injected. 

The progesterone level was checked on the day of HCG injection. Oocyte pickup was performed 36-38 h later. After a period of 48 hours, embryo transfer with Cook catheter (Cook catheter OB/GYN spencer JN) was carried out. The count of embryo transfer that depended on the age and quality of embryo was between 2-4. To support of luteal phase progesterone injection, 100 mg per day from the day of puncture of Oocyte was administered. 

Experiment subjects were selected through interview, examination and lab tests. Upon receiving information, all subjects are consented to the experiment study. For each potential progesterone threshold from 0-1.8 ng/ml in increments of 0.1ng/ml. this method is a variation of receiver operating characteristic (ROC) curve analysis (Honely and McNeil, 1982). 

Serum progesterone breakpoints were optimized by summing the true positive rate and the true negative rate. According to serum progesterone on the day of HCG injection; the patients were divided to two groups based on progesterone level in HCG day. Clinical pregnancy defined as detecting of fetal heart by ultrasound at 6-8 week of gestation, and Chemical pregnancy rate was defined as only β-HCG<200 IU. The rate of pregnancy was compared between the two groups. 

**Statistical analysis**

The experiment data were then compared against the Fisher's exact test in SPSS version 18. The level of significance was considered based on the criteria of p<0.05. 

## Results

As stated earlier, 76 infertile women with PCOS and tubal factor who were suitable candidates for the IVF program. 38 patients were PCOS and 38 were tubal factor. Each group was divided to two clusters based on progesterone level on the day of HCG injection in IVF cycles. Overall pregnancy rate was 26.3% in PCOD patients and 15.8% in tubal factor group. 

The findings of this experiment are summarized in Table I and II. Also 38 tubal factor patients joined in this study were divided to two groups based on progesterone level on the day of HCG injection in IVF cycles. The findings of this experiment are summarized in table III and IV.

**Table I:** Comparison of chemical pregnancy in PCOS patients according to progesterone level

**Group**	**Chemical pregnancy**		**Total**
	**No (%)**	**Yes (%)**	
Progestrone <1.2	16 (84.21%)	3 (15.79%)	19 (100%)
Progestrone ≥1.2	12 (63.16%)	7 (36.84%)	19 (100%)

Fischer`s Exact test p-value= 0.269.

**Table II:** Comparison of clinical pregnancy in PCOS patients according to progesterone level

**Group **	**Chemical pregnancy**		**Total**
	**No (%)**	**Yes (%)**	
Progestrone <1.2	18 (94.74%)	1 (5.26%)	19 (100%)
Progestrone ≥1.2	15 (78.95%)	4 (21.05%)	19 (100%)

Fischer`s Exact test p-value= 0.340.

**Table III:** Comparison of chemical pregnancy in tubal factor patients according to progesterone level

**Group**	**Chemical pregnancy**		**Total**
	**No (%)**	**Yes (%)**	
Progestrone <1.2	16 (84.21%)	3 (15.79%)	19 (100%)
Progestrone ≥1.2	16 (84.21%)	3 (15.79%)	19 (100%)

Fischer`s Exact test p-value= 0.99.

**Table IV:** Comparison of clinical pregnancy in tubal factor patients according to progesterone level

**Group**	**Clinical pregnancy**		**Total**
	**No**	**Yes**	
Progestrone <1.2	17 (89.47%)	2 (10.53%)	19 (100%)
Progestrone ≥1.2	17 (89.47%)	2 (10.53%)	19 (100%)

Fischer`s Exact test p-value=0.99.

## Conclusion

The effect of progesterone level on the IVF outcome has been an uncertainity area for several years (10). Most studies used an absolute progesterone level on the day of HCG administration as an indicator of premature luteinization (PL) and the cutoff level differed from 0.8-2 ng/mL. Some authors defined PL as a progesterone/esteradiol ratio of >1. 

There is a marked variation in the incidence (13-71%), of PL due to discrepancies in definition, population characteristics and/or treatment protocols. The pathogenesis of PL in COH is not well understood. There are some hypotheses explain this subject: rise of follicular LH levels, serum accumulation of HCG from HMG, increased LH receptor sensitivity of the granulosa cells to FSH, or poor ovarian response with increased LH sensitivity. The role of this premature rise of serum progesterone on IVF outcome remain unclear (11).

In the present study, we have compared patients with PCOS and control patients (tubal factor) who had normal ovaries and assessed their pregnancy rate and effect of serum progesterone level on pregnancy rate. Total pregnancy rates were 26.3% in patients with fallopian factor infertility and 15.8% in women with PCOS. In women with PCOS, the pregnancy rate was less in patients with progesterone level <1.2 ng/ml. However this difference was not statistically significant. Likewise, we did not observe any significant differences in pregnancy rate in between two groups of patients with fallopian tube factor infertility. 

Several authors did not find any negative effect of this on IVF outcome (12, 13). In two separate studies, Hoffman and coworkers (7, 8) observed no significant difference in pregnancy rate in patients undergoing IVF/ET with high or low progesterone concentration on the day of HCG administration and in patients who received oocytes donated from women with high or low progesterone concentrations. Huang *et al* (9) showed that there were no differences in clinical pregnancy rate between 3 groups of patients with progesterone level less than 0.3 ng/ml, between 0.3 and 1 ng/ml, and more than 1 ng/ml on the day of HCG injection in IVF cycles. 

Silverberg and coworkers (5) showed that increasing of serum progesterone level on the day of HCG injection had no adverse effect on the quality of Oocytes. Abuzeid *et al* (14) showed that level of progesterone on the day of HCG injection had no negative effect on pregnancy rate. Bustillo *et al* (15) cited that the level of progesterone on the day of HCG injection could not predict pregnancy rate. Lergo *et al* (8) presented PL with serum progesterone higher than 1.2 ng/ml increased pregnancy rate per embryo in donor cases. They also showed that PL with high level progesterone is a common event in IVF cycles. 

It is a natural sign of follicular development; therefore it increases the pregnancy rate. Similarly, Doldi *et al* (4) showed premature increasing of serum progesterone in IVF PCOS cases could be a prediction factor of IVF success. On the contrary however, studies were found that related the PL to low pregnancy rate in IVF cycles. In their study, Schoolcraft and colleagues (16) showed serum progesterone greater than 0.5 ng/ml on the day of HCG injection had significant negative correlation with pregnancy rate in IVF cycles. 

Harada *et al* (17) cited that small increase in serum progesterone on the day of HCG injection had inverse effect on IVF cycles. Burn *et al* (18) proved low pregnancy rate in IVF cycles with premature increase in serum progesterone. Drinfeild and collaborators (19) deduced that the pregnancy rate in IVF cycles was dropped if there was small increase in serum progesterone level on the day of HCG injection. Mio and coworkers (20) also found the same results. These findings suggest that PL may influence the endometrium, adversely affecting implantation and subsequent embryo development due to PL (21).

Based on this information and our results, it seems that premature progesterone production does not have an adverse effect on pregnancy rate, but on the contrary, may be a predictor for success in IVF/ET. In conclusion, to obtain more uniform results, we recommend use of larger samples while the bias variable is taken into account and the ROC curve is used for determination of the unique serum progesterone level.
